# Pilot Study: Comparison of Sourdough Wheat Bread and Yeast-Fermented Wheat Bread in Individuals with Wheat Sensitivity and Irritable Bowel Syndrome

**DOI:** 10.3390/nu9111215

**Published:** 2017-11-04

**Authors:** Reijo Laatikainen, Jari Koskenpato, Sanna-Maria Hongisto, Jussi Loponen, Tuija Poussa, Xin Huang, Tuula Sontag-Strohm, Hanne Salmenkari, Riitta Korpela

**Affiliations:** 1 Faculty of Medicine, Pharmacology, Medical Nutrition Physiology, University of Helsinki, 00014 Helsinki, Finland; hanne.salmenkari@helsinki.fi (H.S.); riitta.korpela@helsinki.fi (R.K.); 2 Aava Medical Centre, 00100 Helsinki, Finland; jari.koskenpato@aava.fi; 3 Fazer Group, 01230 Vantaa, Finland; sanna-maria.hongisto@fazer.com (S.-M.H.); jussi.loponen@fazer.com (J.L.); 4 STAT Consulting Ltd., 37130 Nokia, Finland; tpoussa@netti.fi; 5 Department of Food and Environmental Sciences, University of Helsinki, 00014 Helsinki, Finland; xin.huang@helsinki.fi (X.H.); tuula.sontag-strohm@helsinki.fi (T.S.-S.)

**Keywords:** irritable bowel syndrome, wheat sensitivity, gluten sensitivity, wheat, ATI, amylase trypsin inhibitor, FODMAP

## Abstract

Many patients suspect wheat as being a major trigger of their irritable bowel syndrome (IBS) symptoms. Our aim was to evaluate whether sourdough wheat bread baked without baking improvers and using a long dough fermentation time (>12 h), would result in lower quantities of alpha-amylase/trypsin inhibitors (ATIs) and Fermentable, Oligo-, Di-, Mono-saccharides and Polyols (FODMAPs), and would be better tolerated than yeast-fermented wheat bread for subjects with IBS who have a poor subjective tolerance to wheat. The study was conducted as a randomised double-blind controlled 7-day study (*n* = 26). Tetrameric ATI structures were unravelled in both breads vs. baking flour, but the overall reduction in ATIs to their monomeric form was higher in the sourdough bread group. Sourdough bread was also lower in FODMAPs. However, no significant differences in gastrointestinal symptoms and markers of low-grade inflammation were found between the study breads. There were significantly more feelings of tiredness, joint symptoms, and decreased alertness when the participants ate the sourdough bread (*p* ≤ 0.03), but these results should be interpreted with caution. Our novel finding was that sourdough baking reduces the quantities of both ATIs and FODMAPs found in wheat. Nonetheless, the sourdough bread was not tolerated better than the yeast-fermented bread.

## 1. Introduction

Functional gastrointestinal disorders (FGDs) are common, and irritable bowel syndrome (IBS) is one of the most typical forms, affecting 11% of the adult population in developed countries [1]. IBS patients typically report intolerance to wheat, milk, onion, garlic, apples, alcohol, coffee, chili, and beans [2]. Non-coeliac gluten sensitivity, also known as wheat sensitivity, has attracted attention, both in the popular media and scientific literature [3,4,5,6]. Gluten per se has been long postulated to cause symptoms in some IBS patients, although 86% of patients with subjective wheat tolerance do not seem to respond to gluten pills in masked conditions [7], thus leaving the role of gluten unclear.

A randomised 7-day study concluded that gluten, in low or high doses, did not worsen symptoms of IBS when compared to a whey protein-based placebo in low-FODMAP diet conditions [8]. Indeed, it has been acknowledged that it is unlikely that gluten alone is responsible for all wheat-related symptoms [9]. FODMAPs (Fermentable, Oligo-, Di-, Mono-saccharides and Polyols), i.e., rapidly fermented unabsorbed short-chain carbohydrates in wheat, can cause symptoms in the presence of typical IBS-related features, such as impaired handling of intestinal gas, abnormalities in bowel motility and visceral sensitivity [9,10]. The rapid fermentation of FODMAPs is suggested as a mechanism that triggers IBS symptoms.

Preliminary in vitro and animal studies have indicated that amylase-trypsin inhibitors (ATIs) and other non-gluten proteins have a pro-inflammatory effect on intestinal epithelial cells and thus are theoretically capable of triggering gastrointestinal symptoms in humans [11,12,13]. However, there have been no randomised trials investigating ATIs. In fact, there is an on-going debate as to whether it is wheat proteins (such as ATIs and gluten) or FODMAPs that are the true triggers of wheat-related symptoms in FGD, or if their combination through a possible synergic effect is required for symptom aggravation.

One method of decreasing the amount of FODMAPs and possibly the bioactivity of ATIs in bread (and thus theoretically improving its tolerability), is the utilization of prolonged fermentation processes in breadmaking. Sourdough fermentation activates some proteolytic and fructosidase enzymes in dough, and can thus decrease the amount of proteins and fructans in the end products [14,15,16,17]. The FODMAP content of sourdough bread can be reduced by up to 90% by utilizing a specific sourdough system, as demonstrated in our previous study [16]. Taken together, sourdough baking could theoretically result in wheat products with improved gastrointestinal tolerability.

Recent studies of subjective wheat sensitivity and IBS have suggested that gluten-free and low-FODMAP diets both reduce markers of low-grade inflammation [18,19]. Furthermore, one clinical study showed that kamut wheat, which is an ancient variant of modern wheat, has the potential to reduce inflammatory parameters in blood, as well as other symptoms, when compared to modern wheat [20]. Kamut wheat is lower in ATIs and FODMAPs than modern wheat [13,16].

The present study aimed to evaluate whether a sourdough wheat bread could reduce the symptoms of subjective wheat sensitivity and low-grade inflammation in IBS patients when compared to regular toast bread, baked with added gluten, larger amounts of baker’s yeast, and baking additives. Therefore, these effects were compared in a randomised, double-blinded, parallel group study, while patients were adhering to an otherwise gluten-free diet. We also aimed to specify the ATI and FODMAP content of the two breads.

## 2. Materials and Methods

### 2.1. Subjects and Study Design

A total of 26 patients with IBS were recruited via the internet and from individuals attending a private hospital clinic (Aava Medical Centre, Helsinki, Finland). The eligibility inclusion criteria were as follows: (1) subjects aged 18–65 years; (2) presence of IBS, as defined by the Rome III criteria; and (3) subjective intolerance to wheat.

The diagnosis of subjective wheat intolerance was based on the answer “yes” to the written screening question “I often experience different symptoms when I eat foodstuffs containing wheat and these symptoms are unpleasant”. Intolerance was confirmed by the study dietician during a telephone interview. The headline of the recruitment advertisement was also presented as the question: “Do you experience symptoms if you eat wheat?” We did not perform any blinded exposure tests for wheat, or any biochemical tests, as these do not exist for the diagnosis of wheat intolerance. The exclusion criteria included the presence of an organic gastrointestinal (GI) disease (such as inflammatory bowel disease or celiac disease), major abdominal surgery, any malignancy, pregnancy or breastfeeding, or use of medication potentially influencing gastrointestinal function.

Study candidates were pre-screened by a study dietician. Those subjects meeting the preliminary inclusion criteria were subsequently referred to a screening consultation to a gastroenterologist, who then assessed their final eligibility for participating in the trial. Blood count, sedimentation rate, transglutaminase antibodies for celiac disease, and thyroid function tests were performed during the physician’s screening visit, if these tests had not already been performed within the past 12 months. Laboratory tests were conducted to ensure that the patients were eligible to participate in this trial; for example, all participants with elevated anti-tissue transglutaminase immunoglobulin A (IgA) antibodies were excluded. Patients were provided with obligatory written informed consent forms, which they all signed.

The study protocol was approved by the ethics committee of the Hospital District of Helsinki and Uusimaa. The trial was registered at the United States (US) National Institutes of Health (ClinicalTrials.gov) with the trial number, NCT02572908.

This study was a randomised, double blinded, parallel-group study with a 1-week run-in period, followed by a 1-week treatment period, in which the patients received either the sourdough wheat bread or yeast-fermented wheat bread. Subjects were advised to adhere to a gluten-free diet during the 2-week study duration, the details of which were described to participants by the study dietician. A gluten-free diet was recommended, in order to eliminate any potential confounding, attributable to other gluten-containing grains. The participants were asked to keep 3-day food records during the run-in and treatment period. Randomisation was performed in blocks of four, with a non-concealed allocation. Our statistician generated the computer-assisted randomisation list. The randomisation was blinded for both the participants and the investigators.

### 2.2. Study Breads

The breads were manufactured by Fazer Bakeries (Vantaa, Finland). Both test breads were made using the same baker’s wheat flour (Fazer Mills, Lahti, Finland). The yeast-fermented bread was prepared in an industrial scale bakery, using a straight-dough bread making process that lasted approximately 2 h (including dough mixing, dough rest, moulding, proofing, and oven baking). The recipe of yeast-fermented bread contained added wheat gluten (2% of flour weight), bread improvers (such as emulsifiers), and sorbic acid (E200) as a preservative. The sourdough bread was produced in pilot scale, using a dough mixer and moulded by hand. The sourdough bread used wheat sourdough in the recipe with a long bulk fermentation stage. Overall, the dough stage took more than 12 h. No improvers or any added gluten or preservatives were used in the sourdough bread recipe.

The breads were packaged in white plastic pouches so that participants could not compare the appearance of the study breads (Supplementary Materials Figure S1). The nutrient composition was analysed by Eurofins Scientific Finland (Food & Agro, Raisio, Finland). The dietary fibre content of the breads was determined by using the American Association of Analytical Chemists (AOAC) 2011.25 method [21], a procedure that can discriminate between soluble and insoluble as well as between low and high molecular weight dietary fibres. Resistant starch was identified using the AOAC 2002.02 method [22]. The fructan content was measured by using the AOAC 999.03 method [23] (Megazyme assay kit K-FRUC, Megazyme International Ireland Ltd., Bray, Ireland). The nutritional composition of the study breads is presented in Table 1. The fructan content of sourdough bread represented more than a quarter (26%) of the fructan content of yeast-fermented bread, whereas the protein content was almost the same (94%) as in yeast-fermented wheat bread.

Patients were advised to consume 150 g (six slices) of the study breads every day. Adherence to bread consumption was evaluated by patient entries into a tick-box diary for the treatment period and also by food records.

### 2.3. Determination of Monomeric, Dimeric and Tetrameric ATIs

Sourdough bread and yeast-fermented bread were cut into small pieces, excluding the crusts. Albumins were extracted from 1 g of bread and flour samples, using a 20 mL buffer, containing 10 mM Tris-HCl and 0.1 M ethylenediaminetetraacetic acid (EDTA), with pH 7.5 at 4 °C for 1 h, with gentle shaking. Following centrifugation at 10,000× *g* at 4 °C, the supernatant was collected and precipitated with 50% ammonium sulphate at room temperature. The precipitates were dissolved in the same buffer and its protein concentration was determined by a detergent compatible (DC) protein assay Kit (5000111, Bio-Rad Laboratories Inc., Hercules, CA, USA) with a bovine serum albumin standard. The protein concentration of albumins from sourdough bread, yeast-fermented bread, and flour were adjusted to the same level. The albumin composition was analysed by SDS-PAGE with 12% Bis-Tris protein gel (NP0341BOX, ThermoFisher Scientific, Waltham, MA, USA). The albumin solutions were mixed with a lithium dodecyl sulfate (LDS) sample buffer (NP0008, ThermoFisher Scientific, Waltham, MA, USA), and with or without sample-reducing agent (NP0009, ThermoFisher Scientific, Waltham, MA, USA), and then boiled for 3 min. The running buffer was 3-Morpholinopropane-1-sulfonic acid (MOPS, NP0001), and the protein standard was SeeBlue Plus2 (LC5925, ThermoFisher Scientific, Waltham, MA, USA). The applied voltage was 200 V, and the run time was 50 min. The gel was stained with Coomassie R-250 brilliant blue. The monomer, dimer, and tetramer forms of ATI were recognised according to their molecular weights [24].

### 2.4. Inflammatory Markers

Inflammatory biomarkers, interleukin 8 (IL-8), interleukin 6 (IL-6) and lipopolysaccharide binding protein (LBP), were measured at baseline and after the treatment period with study breads. Serum IL-6 (BMS213HS, ThermoFisher Scientific, Waltham, MA USA), IL-8 (HS800, R&D Systems, Abingdon, UK) and LBP (HK315-01, LBP; Hycult Biotech, Uden, The Netherlands) levels were quantified using sensitive enzyme-linked immunosorbent assays (ELISA).

### 2.5. Symptoms

We constructed a questionnaire based on visual analogue scales (VAS 0–100 mm) to assess the severity of gastrointestinal symptoms and the other symptoms which could be related to IBS. Gastrointestinal symptoms were flatulence, bloating, diarrhoea, constipation, abdominal pain and cramps, borgorygmia, heartburn, nausea, dyspepsia, feeling of incomplete defecation, and urgency in defecation. Other symptoms were tiredness, joint symptoms, skin rash, decreased alertness, and loss of appetite. We did not formally validate the questionnaire with respect to the 17 different symptoms but it follows the practice and concept described by Francis et al. [25] and has been used previously [7,8,17].

### 2.6. Statistical Analysis

No sample size calculations were performed because of the pilot nature of the study and because no benchmark studies existed at the time of the initiation of the study. The patient characteristics are expressed as medians (range) for continuous variables and as number of patients (%) for categorical variables.

Dietary intakes were measured during the run-in period and during the study breads. The Mann–Whitney U test was used to compare the study groups with respect to dietary intakes. The number of consumed slices of study breads was assessed every day during the treatment period. The Mann–Whitney U test was used to compare the average number of consumed slices of study breads. Dietary intakes are expressed as medians (inter-quartile range), due to skewed distributions.

In regard to inflammatory markers, the study breads were compared using an analysis of covariance (ANCOVA), where the baseline was included as a covariate. The distributions of IL-8 and IL-6 were skewed to the right and were logarithmically (ln) transformed before analysis. Due to logarithmic transformation, the comparisons are given as ratios of sourdough bread/yeast-fermented bread.

Gastrointestinal symptoms and the other symptoms were measured daily using VAS (0–100 mm) during the 7-day run-in period and during the 7-day treatment period with study breads. The run-in period was considered the baseline. The symptom scores (mean of weekly measurements) for all symptoms were calculated for baseline and for the treatment period. The total symptom scores were calculated for gastrointestinal symptoms (mean of 12 symptoms) and for other symptoms (mean of five symptoms). Analysis of covariance (ANCOVA) was used when the symptom scores were compared between the study breads and the symptom score during the baseline was included as a continuous covariate. The differences between study breads (sourdough bread vs. yeast-fermented bread) are given as baseline-adjusted means with 95% confidence intervals. The total score for the 12 gastrointestinal symptoms (gastrointestinal total score in Table 5) was the primary outcome variable and the total score for the five other symptoms (other symptoms total score in Table 6) was the key secondary variable. Other secondary outcome variables included the 12 gastrointestinal symptoms and 5 other symptoms, each individually, and the three inflammatory markers. No adjustment of *p*-values for multiple testing was performed for the secondary outcome variables. This is an unpowered exploratory pilot study and *p*-values should not be over-interpreted. Therefore, adjustment of *p*-values was not appropriate.

The statistical analysis was performed using IBM SPSS Statistics for Windows (versions 22.0 and 23.0, IBM Corp, Armonk, NY, USA). A *p*-value < 0.05 was considered statistically significant.

## 3. Results

### 3.1. Baseline Characteristics

In total, 26 subjects with IBS were recruited into the study and none withdrew during its duration. Table 2 depicts the characteristics of the subjects and Supplementary Materials Figure S2, the flow chart. All subjects, except one, were female (96%). Their median age was 43 (range 21–64) years and median body mass index was 25.0 (range 19.6–37.7) kg/m^2^. Four subjects (15%) reported minor use of wheat bread even though it evoked some subjective symptoms, and all of them were randomly assigned into the yeast-fermented bread group. More than half of the subjects (58%) reported following a gluten-free diet before study entry: 6/13 subjects in the sourdough and 9/13 in the yeast-fermented group. The rest reported tolerating wheat in minimal amounts occasionally.

### 3.2. Dietary Intakes

Dietary intakes are reported in Table 3. Consumption of the study breads did not cause any statistically significant differences in the subjects’ energy intakes. Furthermore, there were no statistically significant differences in the intakes of fibre, carbohydrates, fat, or protein between the breads, even although the median fat intake between sourdough bread vs. yeast-fermented bread appeared to differ (79 g vs. 70 grams; *p* = 0.34). The changes in dietary intakes from baseline were also non-significant between study breads (data not shown).

The median (IQR) intake of fructans from the breads was 0.08 (0.07–0.09) g/day with the sourdough wheat bread. This was significantly less than for the yeast-fermented bread, which yielded a value of 0.31 (0.24–0.3) g/day (*p* < 0.001). There was no significant difference in the median number of bread slices consumed per day, i.e., 5.6 (139 g) for the sourdough bread and 5.4 (136 g) for its yeast-fermented counterpart (*p* = 0.92).

### 3.3. ATIs in the Supplied Breads

**Figure 1:**
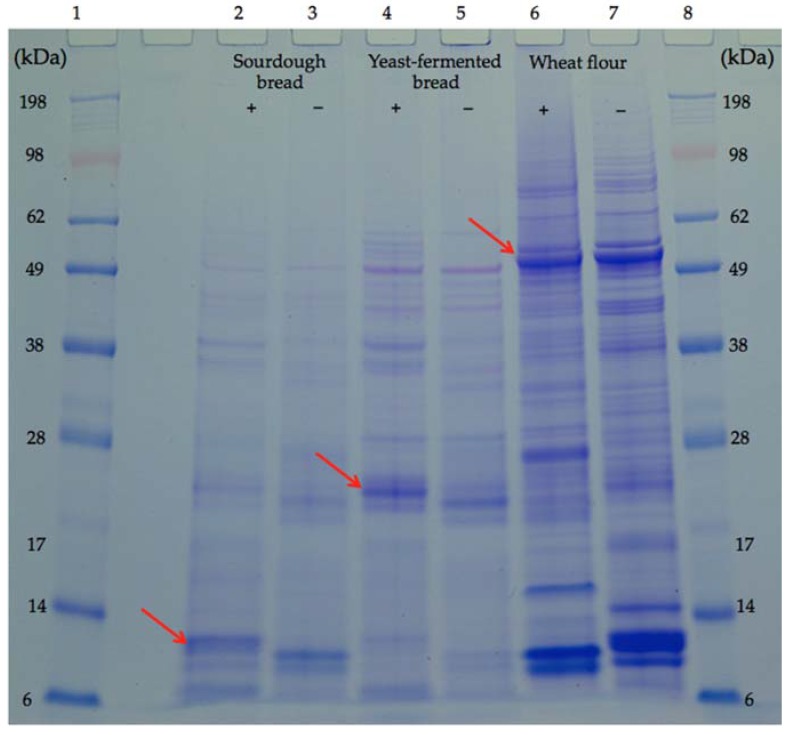
SDS-PAGE of albumins from bread and flour. Lanes 1 and 8: protein molecular weight standard; lanes 2 and 3: albumin extract from sourdough bread; lanes 4 and 5: albumin extract from yeast-fermented bread; lanes 6 and 7: albumin extract from wheat flour. For lanes 2, 4, and 6, marked ‘+’, samples were reduced with dithiothreitol, and those marked ‘−’ were without a reduction. Arrows indicate α-amylase/trypsin inhibitors (ATIs) in different forms, monomer in lane 2, dimer in lane 4 and tetramer in lane 6. kDa: kilodalton.

Both bread samples showed fewer albumin bands than the flour sample, although the protein concentration was the same (Figure 1). The forms of ATI were identified on the basis of their molecular weight [24]. The band intensity of the tetrameric ATIs decreased in the order: flour > yeast-fermented bread > sourdough bread (Figure 1). The dimeric ATI bands in the flour sample were not the same as in the bread samples, and the intensity of these bands in the yeast-fermented bread was higher than in the sourdough bread (Figure 1). The intensity of the monomeric ATI bands decreased in the order: flour > sourdough bread > yeast-fermented bread (Figure 1). These results indicate that compared with the yeast-fermented bread, in the sourdough-fermented bread, the ATIs possibly unravelled from a tetrameric and dimeric form to a monomeric form, more intensively.

### 3.4. Inflammatory Markers

We did not find significant differences between the study breads in the inflammatory markers (Table 4). Furthermore, the changes in inflammatory markers from baseline to the end of treatment period were not significant.

### 3.5. Symptoms

**Figure 2:**
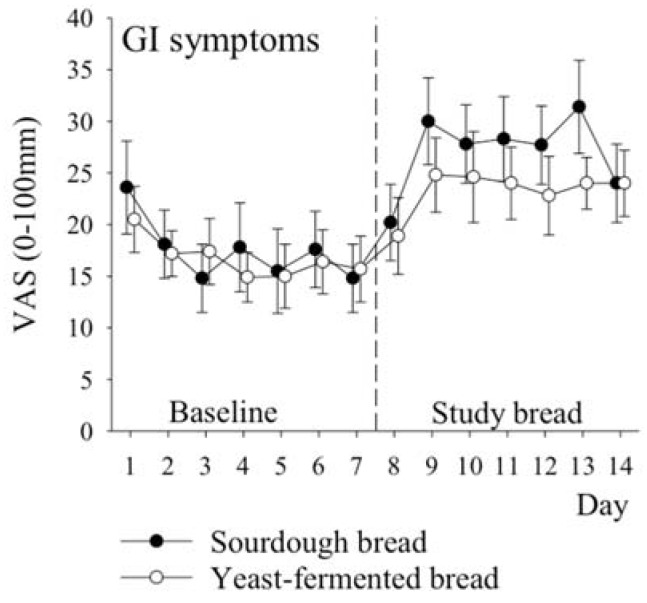
The development of total scores for gastrointestinal symptoms (VAS 0–100 mm) over the course of the study. Days 1–7 (strict gluten free diet) are considered as baseline and days 8–14 as the actual treatment period with study breads. Circles with error bars indicate means (standard error of mean, SEM).

**Figure A1:**
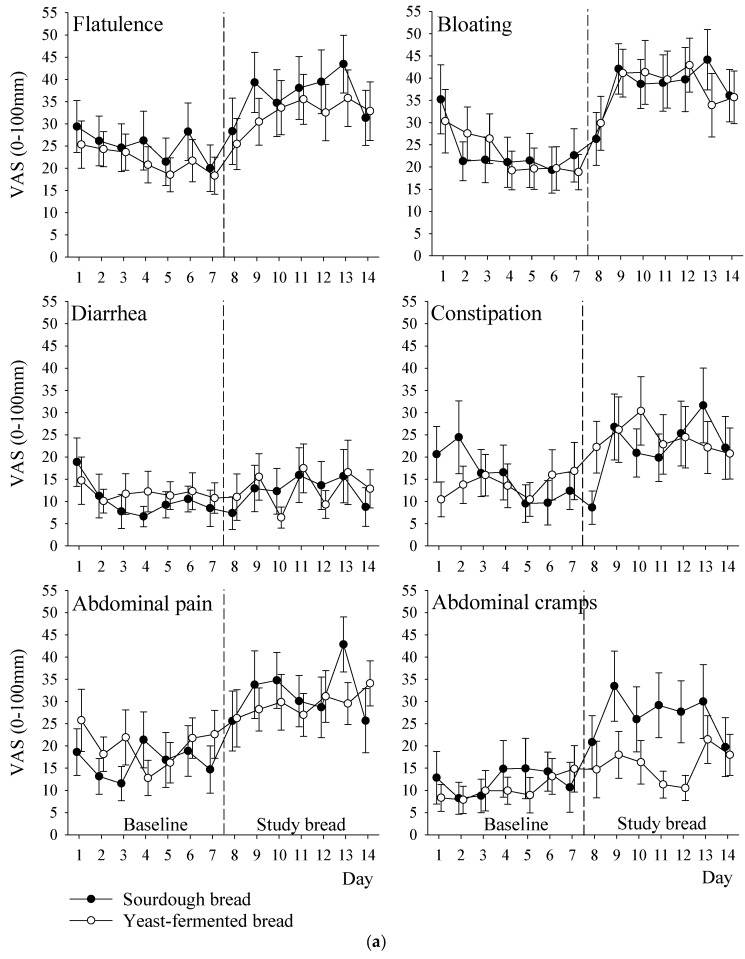

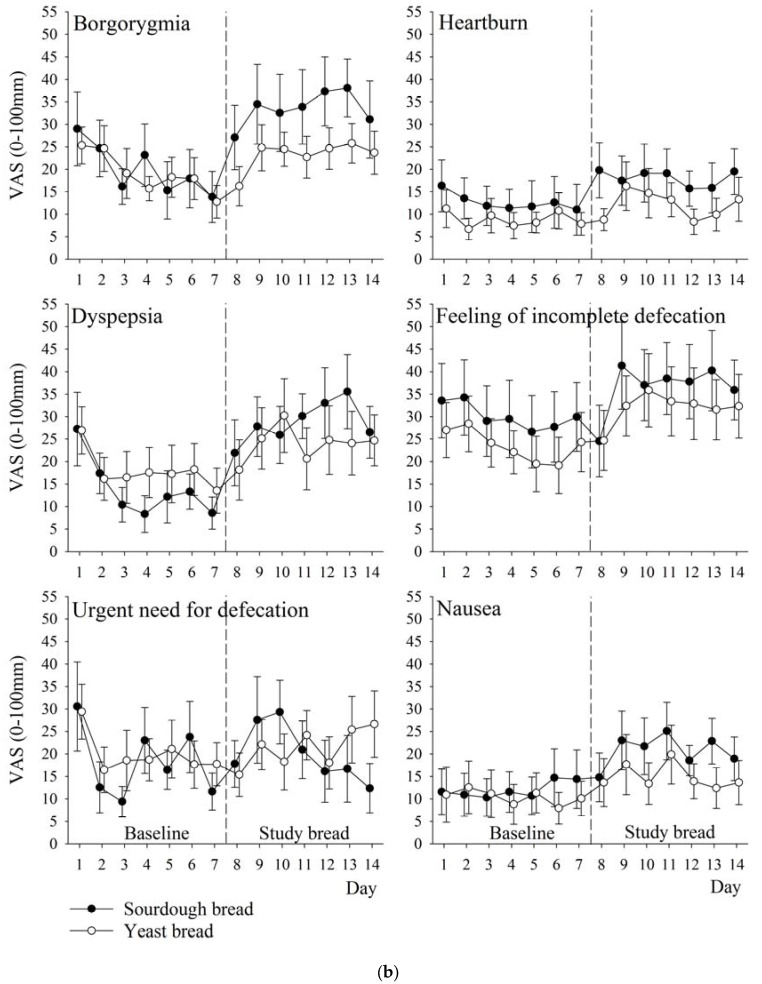
The development of gastrointestinal symptoms (VAS 0–100 mm) over the course of study. Days 1–7 (strict gluten free diet) are considered as the baseline and days 8–14 as the actual treatment period with study breads. Circles with error bars indicate means (SEM). The symptoms are (**a**) flatulence, bloating, diarrhea, constipation, abdominal pain and abdominal cramps, and (**b**) borgorygmia, heartburn, dyspepsia, feeling of incomplete defecation, urgent need for defecation and nausea.

**Figure A2:**
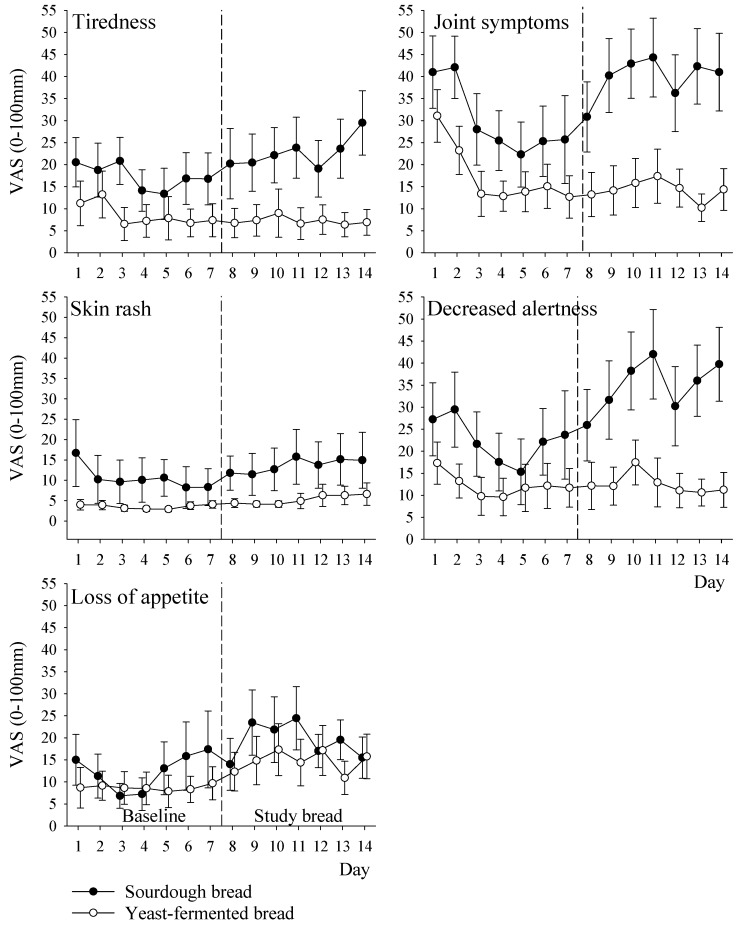
The development of other symptoms (VAS 0–100 mm) over the course of the study. Days 1–7 (strict gluten free diet) are considered as the baseline and days 8–14 as actual treatment period with study breads. Circles with error bars indicate means (SEM).

VAS measurements of gastrointestinal symptoms did not reveal any statistically significant differences between the breads, when the weekly means were analysed (Table 5). However, tiredness, joint symptoms, and decreased alertness were more intense when the subjects were eating the sourdough bread (Table 6, *p*-values: 0.01, 0.03 and 0.003, respectively). There was no significant difference between the breads in terms of the total gastrointestinal symptom score (i.e., the mean of 12 symptoms) which was 27 (Standard deviation, SD 12) mm for the sourdough bread vs. 23 (SD 11) mm for the yeast-fermented bread. The baseline-adjusted difference between breads was 4 (95% CI: −4 to 11) mm, *p* = 0.33. There was a significant difference between the breads for the total non-gastrointestinal score, i.e., in other symptoms, the mean of five symptoms was 26 (SD 18) mm for the sourdough bread vs. 11 (SD 10) mm for the yeast-fermented bread. The baseline-adjusted difference between breads was 8 (95% CI: 2 to 14) mm, *p* = 0.02. The development of the symptoms during both the run-in period (days 1–7) and the treatment period (days 8–14) is illustrated in Figure 2 and in Appendix A (Figure A1a,b and Figure A2).

## 4. Discussion

Our study provides some novel findings. As far as we know, we have demonstrated for the first time that ATI profiles of sourdough and yeast-fermented bread may differ. The molecular weights of the ATIs in this study are in agreement with published values of 60 kDa for tetramers, 24 kDa for dimers, and 12 kDa for monomers [24]. The lower content of ATI polymers (molecular weight >14 kDa) in the sourdough bread than in the yeast-fermented bread is likely attributable to the more intensive reduction during sourdough fermentation, as shown by the greater intensity of the monomeric ATI bands in the sourdough lanes. Reduced ATIs per se have been reported to have less stimulatory activity against toll-like-receptor 4 (TLR4), and thus cause less innate immune reactions, in vitro and in animals [11,13], but no clinical trials had been performed on ATIs prior to our study.

As far as we know, this is the first study to show that sourdough baking apparently more extensively unravels the tetrameric and dimeric form of ATIs when compared to yeast-fermented bread/wheat flour. This may be due to disulphide bond reduction, as sourdough redox conditions are known to be reductive [26]. The remaining ATIs in sourdough bread had weights of approximately 12 kDa, and thus were likely members of the ATI species, chloroform-methanolsoluble (CM) 0.28 [27]. ATIs of the CM 0.28 family do not have an especially pro-inflammatory effect in the gastrointestinal tract [11,13]. The sourdough bread was also absent of baking improvers (additives), when compared to yeast-fermented bread. Despite being lower in ATIs, fructans, and additives, the sourdough bread caused a rapid increase in many symptoms, and was not tolerated better than the yeast-fermented bread. This was in contrast to our hypothesis; the sourdough bread actually caused more non-gastrointestinal symptoms. We believe that the observed difference in non-gastrointestinal symptoms between the groups is likely to be due to chance, or due to a slight difference between the study groups at baseline (see discussion below). Theoretically, it is possible, albeit unlikely, that some unrecognized factor, such as metabolites in sourdough bread, might have caused the observed difference. The small sample size is a limitation of our study, and needs to be considered in this context. Our symptom evaluation was based on VAS measurements, which are not validated by dietary IBS studies, even if they are widely used. Clearly, further research is warranted to confirm the pilot results reported herein.

Secondly, we demonstrated that the consumption of 5.4–5.6 slices of the wheat breads daily, as a sole source of wheat, did not increase inflammatory markers of innate immune activation, even though symptoms worsened rapidly. Therefore, the observed rapid worsening of symptoms, especially in sourdough bread, is unlikely to be caused by pro-inflammatory effects of wheat in the intestine. On the contrary, we argue that the major driver of the symptoms was the nocebo effect; participants may have expected adverse effects to any kind of wheat products, due to previous experiences and/or beliefs. Alternatively, both wheat breads per se might have caused gastrointestinal symptoms by forming a bolus in stomach, inhibiting gastric emptying, and reducing small bowel water, as a magnetic resonance imaging (MRI) study with whole wheat bread has suggested [28].

Seven days may be too short of a time period to detect changes in inflammatory markers, although previous experimental findings have suggested that pro-inflammatory reactions to wheat can be rapid [13,29]. Our first clinical results related to ATIs and markers of innate immunity urge the need for further studies, in order to clarify the effects of wheat products—such as bread and pasta—on innate immunity. It would be of interest to compare ATI profiles of sourdough wheat bread, wheat pasta and wheat flour. One would assume that pasta is higher in intact ATIs than sourdough bread, as it is not exposed to the fermentation process in food manufacturing. On the other hand, pasta is cooked in hot water and rinsed with water after cooking, which may remove a remarkable proportion of ATIs, as they are water-soluble albumin proteins.

Finally, the FODMAP content of our sourdough wheat bread was reduced by 74%, as compared to yeast-fermented bread (fructans 0.23 g/100 g), making our sourdough wheat bread clearly lower in fructans (0.06 g/100 g) than rice cakes or rice bubbles [30]. Rice is known to be very low in fructans and well tolerated. Consequently, the remaining FODMAPs in sourdough wheat bread are unlikely triggers of symptoms unless bread is eaten in extreme portions per sitting. We also hasten to add that our results should not be interpreted as a critique against holistic low-FODMAP diets in the treatment of IBS, simply because the achieved daily reduction of intake of fructans was likely too small in total. The achieved difference in the FODMAP intake due to the breads was approximately 0.3 g/day, and such a difference represents just 20% of what has been shown to be a minimal meaningful difference, i.e., 1.5 g/day [17]. Even if we did not expect the difference in FODMAPs in the breads to play a major role, we hypothesized that the synergic effect of several potentially beneficial changes might play a role; after all, sourdough bread was found to have a lower content of FODMAPs, ATIs, gluten, and additives. Furthermore, it also seems that the yeast-fermented bread we used in our study was lower in FODMAPs than those that are marketed elsewhere, i.e., 0.23 g/100 g in our study compared to 0.68 g/100 g in Biesiekierski’s Australian study [30].

Our study has some limitations. As no clinical ATI studies were available at the time of the initiation of the study, we did not perform power calculations. A slight difference between the groups in the tolerance of wheat at baseline (Table 2) might have caused some bias into symptoms, favouring yeast-fermented bread; nevertheless, no differences were seen in the inflammatory markers at baseline, nor after the treatments, suggesting that any potentially existing bias was marginal. Finally, we did not compare in vitro inflammatory responses to ATIs of our study products and therefore were unable to quantify the theoretic inflammatory potential of the wheat products used—we are considering this in the future, in order to confirm our novel findings. Moreover, ATIs observed in this study were not identified by sequencing or immunological methods and must thus be considered likely ATIs.

A crossover design might have been a more appropriate choice for the purpose of this study, rather than the parallel group design. However, there were two reasons we did not want to run a crossover study. Firstly, we considered that many patients might drop out from the study if they had to expose to themselves to a substantial amount gluten/wheat breads (approximately 150 g/day) during the separate periods, as they would have in a crossover design. Furthermore, the sourdough wheat bread and yeast-fermented wheat bread had different physical appearances (supplemental image). If all participants had been exposed to both breads, the key strength of our study—blinding—might have been lost. Participants might have guessed by appearance, which bread was sourdough, and which one was yeast-fermented. For these reasons we chose a parallel design. We emphasise that our pilot results must be considered a preliminary effort, paving the way for larger studies, and we recognise that the number of participants in our study was low for a parallel group design.

## 5. Conclusions

In conclusion, our pilot study did not reveal any significant differences in gastrointestinal tolerability or in low-grade inflammation when comparing sourdough wheat bread and yeast-fermented wheat bread, in subjects with irritable bowel syndrome/poor tolerance to wheat. Our findings, together with those of recent clinical trials on wheat sensitivity [31], underline the need for larger-scale clinical studies. Data from in vitro and animal studies cannot be extrapolated into clinical care and as such randomised studies are needed to verify pre-clinical outcomes. Furthermore, our study also highlights the fact that ATI analyses of wheat flour or other unfermented wheat products, cannot be extrapolated to fermented wheat products, such as sourdough bread. More studies with longer treatment periods and larger sample sizes are required to further clarify the role of ATIs and grain products as such in functional gastrointestinal disorders. The nocebo response seems to play a major role in IBS and subjective wheat sensitivity, and might explain our findings regarding non-gastrointestinal symptoms. The nocebo effect should be carefully addressed when planning future studies.

## Figures and Tables

**Table 1:** Nutritional composition of the study breads.

	Sourdough Bread */100 g	Yeast-Fermented Bread */100 g
Energy, kJ (kcal)	972 (229)	1116 (264)
Protein, g	9.3	9.9
Gluten, g	4.8	6.0
Fat, g	1.2	2.4
Carbohydrates, g	43.4	48.4
Dietary fibre, g	3.9	4.5
Salt, g	1.2	1.1
Fructans, g	0.06	0.23
LMWDF, g	0.9	1.0
Insoluble HMWDF, g	1.8	2
Soluble HMWDF, g	1.2	1.4
Resistant starch, g	0.7	0.8
Dominant ATI form	Monomeric	Dimeric
Additives/Improvers	Not present	Present

LMWDF: Low molecular weight dietary fibre; HMWDF: High molecular weight dietary fibre; ATI: alpha-amylase/trypsin inhibitor. * Participants were supplied with and instructed to consume 150 grams of each bread/day.

**Table 2:** Baseline characteristics of the participants.

Characteristics	Sourdough Bread (*n* = 13)	Yeast-Fermented Bread (*n* = 13)	Total (*n* = 26)
Females	12 (92)	13 (100)	25 (96)
Age (years)	39 (21–56)	47 (24–64)	43 (21–64)
Body mass index, BMI (kg/m^2^)	25.1 (20.2–37.7)	24.9 (19.6–31.8)	25.0 (19.6–37.7)
Weight (kg)	70 (59–109)	65 (54–92)	68 (54–109)
Self-reported daily consumption of any bread (number of slices)	3.0 (1–4)	2.5 (1–7.5)	3.0 (1–7.5)
Occasional use of some wheat bread	0 (0)	4 (31)	4 (15)

Results are given as median (range) and as *n* (%) of subjects.

**Table 3:** Dietary intake during the baseline and during consumption of the study breads.

		Sourdough Bread (*n* = 13)	Yeast-Fermented Bread (*n* = 13)	*p*-Value *
Energy, kJ/day	Baseline	8422 (7521–9530)	8177 (7062–8425)	0.80
	Study bread	8214 (7826–8558)	7609 (6498–9899)	
Energy, kcal/day	Baseline	2012 (1797–2276)	1953 (1687–2012)	0.84
	Study bread	1962 (1869–2044)	1818 (1553–2365)	
Carbohydrates, g/day	Baseline	196 (147–236)	182 (161–215)	0.88
	Study bread	198 (169–221)	181 (166–281)	
Protein, g/day	Baseline	88 (64–103)	78 (64–102)	0.36
	Study bread	89 (85–107)	85 (64–107)	
Fat, g/day	Baseline	89 (66–103)	73 (69–88)	0.34
	Study bread	79 (78–86)	70 (53–83)	
Dietary fibre, g/day	Baseline	20 (15–22)	16 (14–22)	0.36
	Study bread	20 (17–22)	16 (15–24)	

Results are given as median (inter-quartile range). * Dietary intakes during study breads and also the changes from baseline were compared between the study breads with the Mann–Whitney U test. *p*-values for changes from baseline varied between 0.13 and 0.96.

**Table 4:** Interleukin-8 (IL-8; pg/mL), interleukin-6 (IL-6; pg/mL) and lipopolysaccharide binding protein (LBP; µg/mL) at baseline and after the treatment periods with study breads. Confidence intervals (CI) are in parentheses.

	Sourdough Bread (*n* = 12)		Yeast-Fermented Bread (*n* = 12)		Sourdough Bread vs. Yeast-Fermented Bread	
	Baseline	Study Bread	Baseline	Study Bread	Baseline-Adjusted Difference	
					Ratio (95% CI)	*p-*Value *
IL-8 (pg/mL)	11.6 (9.8–13.8)	11.2 (10.1–12.4)	13.7 (11.0–17.0)	13.3 (10.5–16.9)	0.91 (0.73 to 1.14)	0.38
IL-6 (pg/mL)	0.67 (0.34–1.32)	0.72 (0.42–1.21)	1.22 (0.78–1.90)	1.09 (0.73–1.64)	1.01 (0.74 to 1.39)	0.38
					**Mean (95% CI)**	***p-*Value**
LBP (µg/mL)	8.39 (5.16–11.62)	8.48 (5.09–11.88)	8.15 (6.45–9.85)	9.42 (7.15–11.70)	−1.19 (−2.55 to 0.17)	0.08

Results are given as geometric means (95% CI) for IL-8 and IL-6 and as the arithmetic mean (95% CI) for LBP. * The study breads were compared using an analysis of covariance (ANCOVA), where baseline was included as a covariate. IL-8 and IL-6 were logarithmically (ln) transformed before the analysis.

**Table 5:** Gastrointestinal symptoms (visual analogue scales, VAS 0–100 mm) and gastrointestinal total score during the baseline (mean of days 1–7) and during consumption of the study breads (mean of days 8–14).

Symptom	Sourdough Bread (*n* = 13)		Yeast-Fermented Bread (*n* = 13)		Sourdough Bread vs. Yeast-Fermented Bread	
	Baseline	Study Bread	Baseline	Study Bread	Baseline-Adjusted Difference	*p*-Value *
Flatulence	25 (16)	37 (20)	22 (12)	32 (17)	3 (−11 to 17)	0.66
Bloating	23 (17)	38 (16)	23 (15)	38 (20)	1 (−13 to 15)	0.93
Diarrhoea	10 (8)	13 (15)	12 (10)	13 (10)	1 (−9 to 10)	0.90
Constipation	16 (13)	22 (17)	14 (13)	24 (20)	−4 (−16 to 8)	0.54
Abdominal pain	16 (14)	32 (15)	20 (15)	29 (15)	4 (−7 to 15)	0.44
Abdominal cramps	12 (17)	27 (19)	10 (10)	16 (13)	10 (−1 to 21)	0.06
Borgorygmia	20 (20)	34 (24)	19 (12)	23 (13)	10 (−1 to 21)	0.07
Heartburn	13 (16)	18 (16)	9 (7)	12 (10)	3 (−5 to 12)	0.43
Dyspepsia	14 (14)	29 (17)	18 (17)	24 (23)	8 (−6 to 22)	0.25
Feeling of incomplete defecation	30 (25)	37 (25)	24 (16)	32 (24)	−1 (−14 to 13)	0.93
Urgent need for defecation	18 (14)	21 (19)	20 (15)	21 (16)	1 (−10 to 12)	0.90
Nausea	12 (17)	21 (15)	10 (16)	15 (16)	5 (−4 to 13)	0.29
Gastrointestinal total score	17 (12)	27 (12)	17 (9)	23 (11)	4 (−4 to 11)	0.33

Results are given as mean (SD) and mean (95% CI). * The study breads were compared using the analysis of covariance (ANCOVA), where the symptom score during the baseline was included as a covariate.

**Table 6:** Other symptoms (VAS 0–100 mm) and other symptoms total score during the baseline (mean of days 1–7) and during the consumption of the study breads (mean of days 8–14).

Symptom	Sourdough Bread (*n* = 13)		Yeast-Fermented Bread (*n* = 13)		Sourdough Bread vs. Yeast-Fermented Bread	
	Baseline	Study Bread	Baseline	Study Bread	Baseline-Adjusted Difference	*p*-Value *
Tiredness	30 (24)	40 (25)	17 (15)	14 (15)	16 (4 to 27)	0.01
Joint symptoms	17 (19)	23 (21)	9 (14)	7 (13)	7 (1 to 13)	0.03
Skin rash	11 (18)	14 (18)	3.5 (3)	5 (5)	3 (−5 to 11)	0.45
Decreased alertness	22 (25)	35 (26)	12 (15)	13 (12)	14 (5 to 22)	0.003
Loss of appetite	12 (18)	19 (15)	9 (12)	15 (15)	2 (−6 to 9)	0.62
Other symptoms total score	19 (17)	26 (18)	10 (11)	11 (10)	8 (2 to 14)	0.02

Results are given as mean (SD) and mean (95% CI). * The study breads were compared using the analysis of covariance (ANCOVA), where the symptom score during the baseline was included as a covariate.

## Supplementary Materials

The following are available online at www.mdpi.com/2072-6643/9/11/1215/s1, Figure S1: Flow chart, Figure S2: Pouched study breads.

## References

[1] Canavan C., West J., Card T. (2014). The epidemiology of irritable bowel syndrome. Clin. Epidemiol..

[2] Simrén M., Månsson A., Langkilde A.M., Svedlund J., Abrahamsson H., Bengtsson U., Björnsson E.S. (2001). Food-related gastrointestinal symptoms in the irritable bowel syndrome. Digestion.

[3] Hadjivassiliou M., Sanders D.S., Grünewald R.A., Woodroofe N., Boscolo S., Aeschlimann D. (2010). Gluten sensitivity: From gut to brain. Lancet Neurol..

[4] Fasano A., Sapone A., Zevallos V., Schuppan D. (2015). Nonceliac gluten sensitivity. Gastroenterology.

[5] Makharia A., Catassi C., Makharia G.K. (2015). The Overlap between Irritable Bowel Syndrome and Non-Celiac Gluten Sensitivity: A Clinical Dilemma. Nutrients.

[6] Markham H. (2015). You Asked: Do I Have a Gluten Allergy?. Time Magazine.

[7] Elli L., Tomba C., Branchi F., Roncoroni L., Lombardo V., Bardella M.T., Buscarini E. (2016). Evidence for the presence of non-celiac gluten sensitivity in patients with functional gastrointestinal symptoms: Results from a multicenter randomized double-blind placebo-controlled gluten challenge. Nutrients.

[8] Biesiekierski J.R., Peters S.L., Newnham E.D., Rosella O., Muir J.G., Gibson P.R. (2013). Effects of gluten in patients with self-reported non-celiac gluten sensitivity after dietary reduction of fermentable, poorly absorbed, short-chain carbohydrates. Gastroenterology.

[9] De Giorgio R., Volta U., Gibson P.R. (2016). Sensitivity to wheat, gluten and FODMAPs in IBS: Facts or fiction?. Gut.

[10] Gibson P.R., Shepherd S.J. (2010). Evidence-based dietary management of functional gastrointestinal symptoms: The FODMAP approach. J. Gastroenterol. Hepatol..

[11] Junker Y., Zeissig S., Kim S.J., Barisani D., Wieser H., Schuppan D. (2012). Wheat amylase trypsin inhibitors drive intestinal inflammation via activation of toll-like receptor 4. J. Exp. Med..

[12] Valerii M.C., Ricci C., Spisni E., Di Silverstro R., Cavazza E., Dinelli G. (2015). Responses of peripheral blood mononucleated cells from non-celiac gluten sensitive patients to various cereal sources. Food Chem..

[13] Zevallos V.F., Raker V., Tenzer S., Jimenez-Calvente C., Schuppan D. (2017). Nutritional Wheat Amylase-Trypsin Inhibitors Promote Intestinal Inflammation via Activation of Myeloid Cells. Gastroenterology.

[14] Chavan S., Chavan S. (2011). Sourdough technology—A traditional way for wholesome foods: A review. Compr. Rev. Food Sci. Food Saf..

[15] Loponen J., Sontag-Strohm T., Venäläinen J., Salovaara H. (2007). Prolamin hydrolysis in wheat sourdoughs with differing proteolytic activities. J. Agric. Food Chem..

[16] Ziegler J., Steiner D., Longin C., Friedrich H., Würschum T., Schweiggert R., Carle R. (2016). Wheat and the irritable bowel syndrome—FODMAP levels of modern and ancient species and their retention during bread making. J. Funct. Foods.

[17] Laatikainen R., Koskenpato J., Hongisto S.M., Loponen J., Poussa T., Korpela R. (2016). Randomised clinical trial: Low-FODMAP rye bread vs. regular rye bread to relieve the symptoms of irritable bowel syndrome. Aliment. Pharmacol. Ther..

[18] Hustoft T.N., Hausken T., Ystad S.O., Valeur J., Brokstadt K., Hattlebak J.G., Lied G.A. (2017). Effects of varying dietary content of fermentable short-chain carbohydrates on symptoms, fecal microenvironment, and cytokine profiles in patients with irritable bowel syndrome. Neurogastroenterol. Motil..

[19] Uhde M., Ajamian M., Caio G., DiGiorgio R., Indart A., Alaedini A. (2016). Intestinal cell damage and systemic immune activation in individuals reporting sensitivity to wheat in the absence of coeliac disease. Gut.

[20] Sofi F., Whittaker A., Gori A.M., Cesari F., Surrenti E., Casini A. (2014). Effect of Triticum turgidum subsp. turanicum wheat on irritable bowel syndrome: A double-blinded randomised dietary intervention trial. Br. J. Nutr..

[21] McCleary B.V., DeVries J.W., Rader J.I., Cohen G., Prosky L., Mugford D.C., Okuma K.J. (2012). Determination of insoluble, soluble, and total dietary fiber (CODEX definition) by enzymatic-gravimetric method and liquid chromatography: Collaborative study. AOAC Int..

[22] McCleary B.V., Monaghan D.A. (2002). Measurement of resistant starch. J. AOAC Int..

[23] McCleary B.V., Murphy A., Mugford D.C. (2000). Measurement of total fructan in foods by enzymatic/spectrophotometric method: Collaborative study. J AOAC Int..

[24] Gomez L., Sanchez-Monge R., Garcia-Olmedo F., Salcedo G. (1989). Wheat tetrameric inhibitors of insect α-amylases: Alloploid heterosis at the molecular level. Proc. Natl. Acad. Sci. USA.

[25] Francis C.Y., Morris J., Whorwell P.J. (1997). The irritable bowel severity scoring system: A simple method of monitoring irritable bowel syndrome and its progress. Aliment. Pharmacol. Ther..

[26] Loponen J., König K., Wu J., Gänzle M.G. (2008). Influence of thiol metabolism of lactobacilli on egg white proteins in wheat sourdoughs. J. Agric. Food Chem..

[27] Choudhury A., Maeda K., Murayama R., DiMagno E.P. (1996). Character of a wheat amylase inhibitor preparation and effects on fasting human pancreaticobiliary secretions and hormones. Gastroenterology.

[28] Marciani L., Pritchard S.E., Hellier-Woods C., Costigan C., Spiller R.C. (2013). Delayed gastric emptying and reduced postprandial small bowel water content of equicaloric whole meal bread versus rice meals in healthy subjects: Novel MRI insights. Eur. J. Clin. Nutr..

[29] Fritscher-Ravens A., Schuppan D., Ellrichmann M., Schoch S., Milla P.J. (2014). Confocal endomicroscopy shows food-associated changes in the intestinal mucosa of patients with irritable bowel syndrome. Gastroenterology.

[30] Biesiekierski J.R., Rosella O., Rose R., Liels K., Barrett J.S., Muir J.G. (2011). Quantification of fructans, galacto-oligosacharides and other short-chain carbohydrates in processed grains and cereals. J. Hum. Nutr. Diet..

[31] Molina-Infante J., Carroccio A. (2017). Suspected Nonceliac Gluten Sensitivity Confirmed in Few Patients after Gluten Challenge in Double-Blind, Placebo-Controlled Trials. Clin. Gastroenterol. Hepatol..

