# Supplementary material for: Pilot Study: Comparison of Sourdough Wheat Bread and Yeast-Fermented Wheat Bread in Individuals with Wheat Sensitivity and Irritable Bowel Syndrome
Source: Nutrients. 2017 Nov 4;9(11):1215. doi: 10.3390/nu9111215 (PMC5707687; doi:10.3390/nu9111215)
Supplement: Supplementary file 1 [file nutrients-09-01215-s001.pdf]

nutrients

Supplements

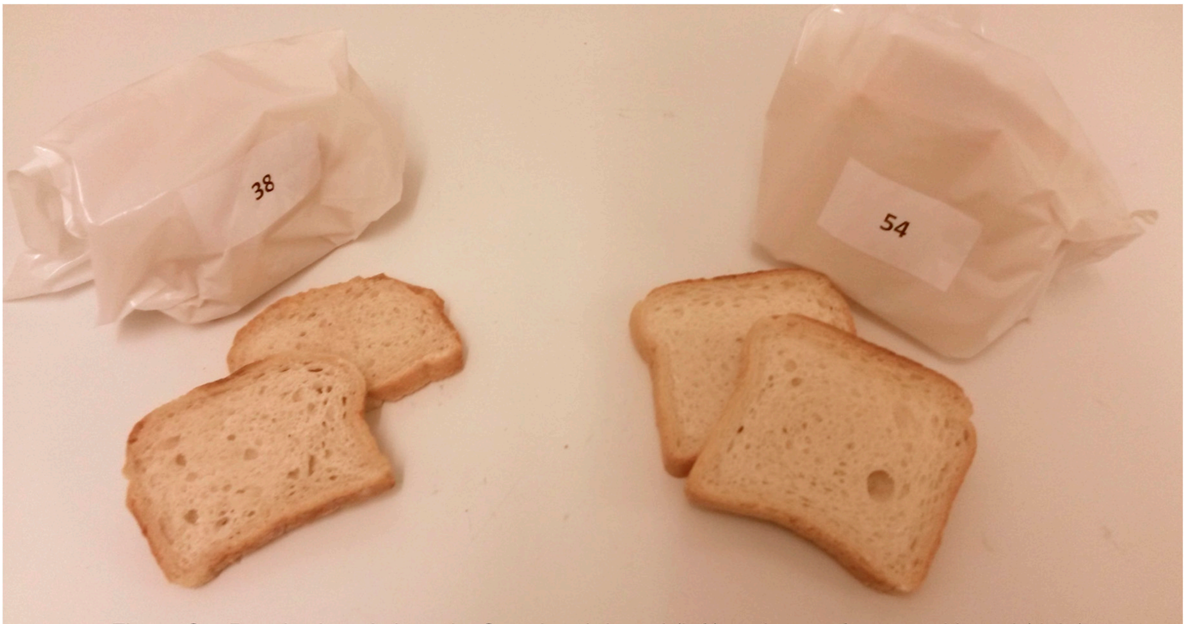

Figure S1. Pouched study breads. Sourdough bread (left) and yeast-fermented bread (right).

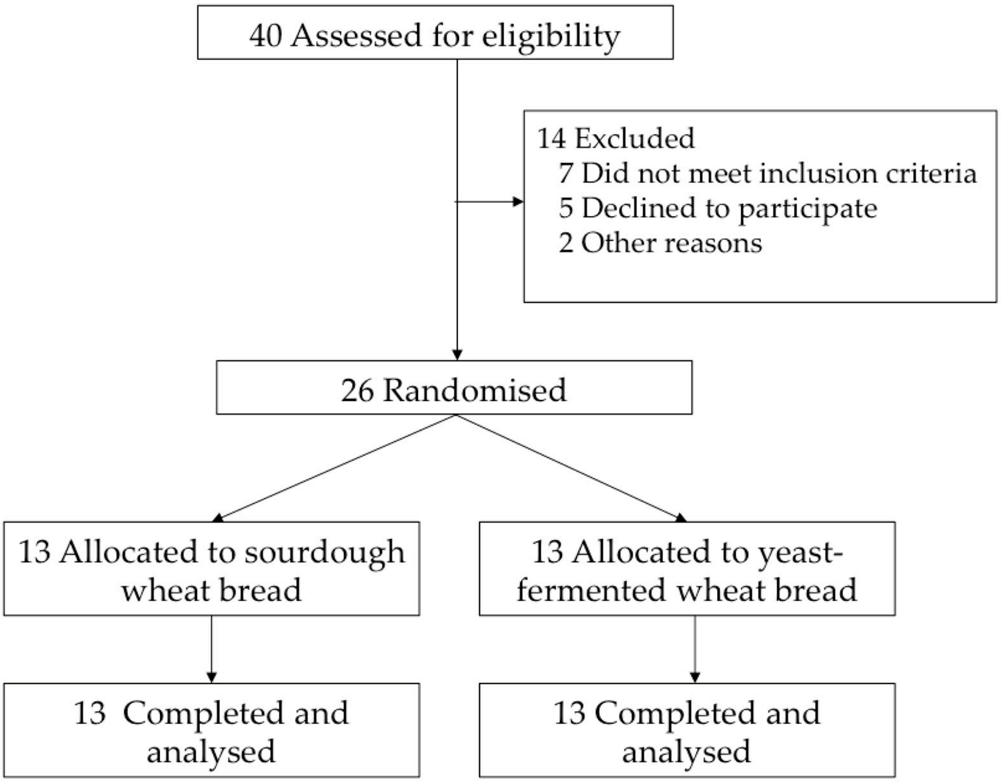

Figure S2. Flow chart

Appendix

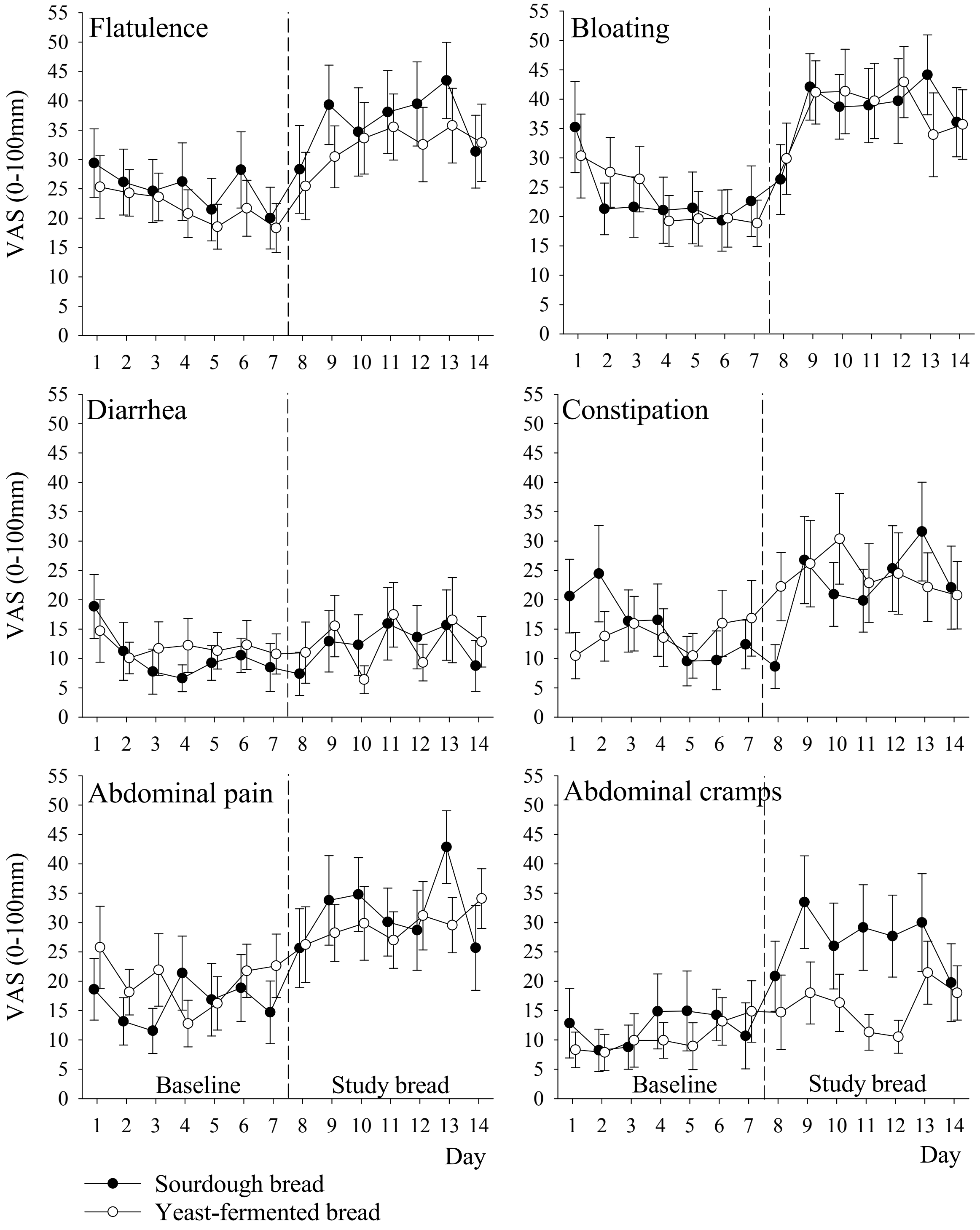

Figure A1a. The development of gastrointestinal symptoms (VAS 0–100 mm) over the course of the study. Days 1–7 (strict gluten free diet) are considered as the baseline and days 8–14 as the actual treatment period with study breads. Circles with error bars indicate means (SEM).

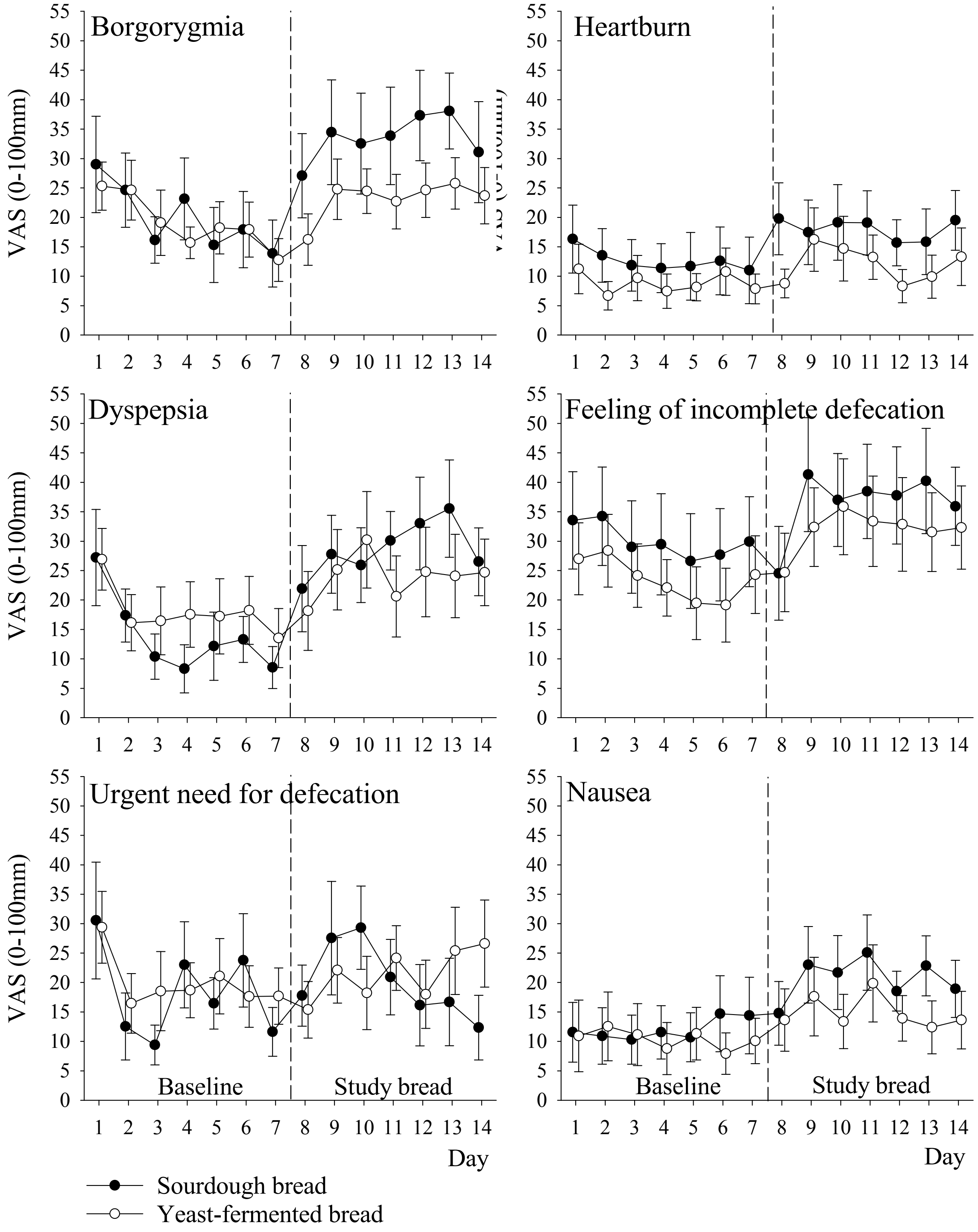

Figure A1b. The development of gastrointestinal symptoms (VAS 0–100 mm) over the course of the study. Days 1–7 (strict gluten free diet) are considered the baseline and days 8–14 as the actual treatment period with study breads. Circles with error bars indicate means (SEM).

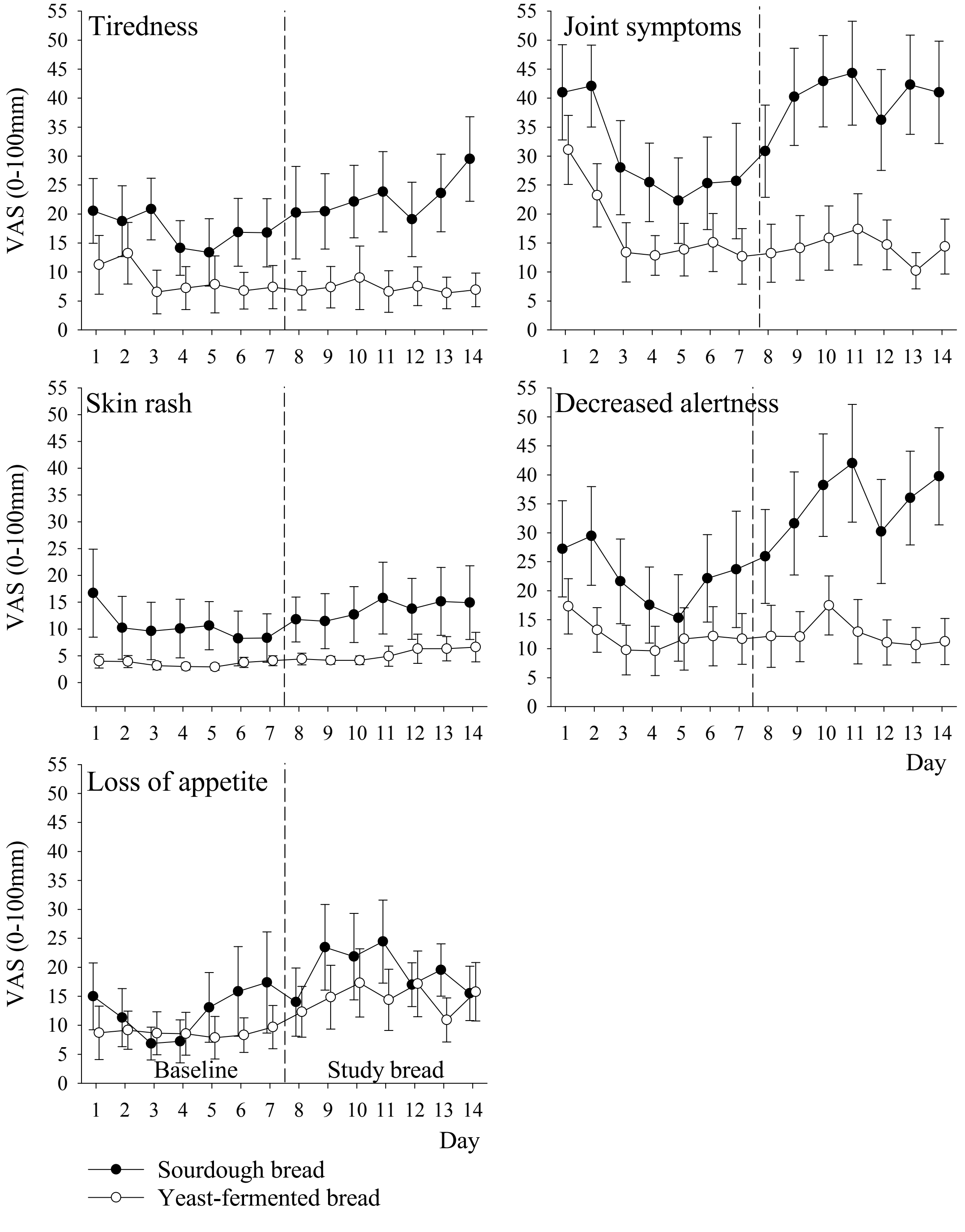

Figure A2. The development of other symptoms (VAS 0–100 mm) over the course of the study. Days 1–7 (strict gluten free diet) are considered as the baseline and days 8–14 as actual treatment period with study breads. Circles with error bars indicate means (SEM).
